# Synergistic effects of intradialytic concurrent training and melatonin supplementation on immune cell adaptations in hemodialysis patients

**DOI:** 10.3389/fphar.2026.1880592

**Published:** 2026-08-28

**Authors:** Houssem Marzougui, Hend Hachicha, Rami Maaloul, Imen Ben Dhia, Salma Toumi, Choumous Kallel, Hanen Maamri, Khaoula Kammoun, Fatma Ayadi, Hatem Masmoudi, Omar Hammouda, Mohamed Ben Hmida

**Affiliations:** 1 Research Laboratory Molecular Bases of Human Pathology LR19ES13, Faculty of Medicine, University of Sfax, Tunisia; 2 EM2S, LR19JS01, High Institute of Sport and Physical Education of Sfax, University of Sfax, Sfax, Tunisia; 3 Immunology Department, CHU Habib Bourguiba, University of Sfax, Tunisia; 4 Research Laboratory, Autoimmunity Cancer and Immunogenetics, LR18/SP12, CHU Habib Bourguiba, University of Sfax, Tunisia; 5 Research Laboratory of Evaluation and Management of Musculoskeletal System Pathologies LR20ES09, Faculty of Medicine, University of Sfax, Tunisia; 6 Nephrology Department, CHU Hedi Chaker, University of Sfax, Tunisia; 7 Research Laboratory of Renal Pathology LR19ES11, Faculty of Medicine, University of Sfax, Tunisia; 8 Hematology Laboratory, CHU Habib Bourguiba, University of Sfax, Sfax, Tunisia; 9 Department of Community Health and Epidemiology, Hedi Chaker University Hospital, University of Sfax, Sfax, Tunisia; 10 Biochemistry Laboratory, CHU Habib Bourguiba, University of Sfax, Sfax, Tunisia; 11 LINP2, UFR STAPS, University of Paris Nanterre, Nanterre, France

**Keywords:** cardiovascular risk, concurrent training, hemodialysis, immune modulation, intradialytic exercise, melatonin, monocyte subtypes, T lymphocytes

## Abstract

**Background:**

Immune dysfunction and chronic low-grade inflammation contribute substantially to morbidity and cardiovascular risk in patients undergoing hemodialysis (HD). Both exercise-based interventions and melatonin supplementation have demonstrated immunomodulatory and anti-inflammatory potential; however, their combined long-term effects remain unclear. This study investigated whether a 12-week intradialytic concurrent training (ICT) program with melatonin supplementation could improve hematological and immune cell profiles in HD patients.

**Methods:**

Thirty HD patients were randomly allocated to ICT with melatonin (ICT-MEL), ICT with placebo (ICT-PLA), or control with placebo (C-PLA). ICT groups trained three times per week. During the intervention, participants received either 3 mg of melatonin (ICT-MEL) or placebo (ICT-PLA and C-PLA) nightly before nocturnal sleep. Pre- and post-intervention blood samples were analyzed for hematological parameters, adaptive lymphocyte subsets (CD4^+^ T cells, CD8^+^ T cells, and B cells), innate lymphocytes (natural killer [NK] cells), and monocyte subsets.

**Results:**

Hematological measures remained stable from pre-to post-intervention across all groups. In contrast, the ICT-MEL intervention induced significant within-group improvements in T-cell immunity, including increases in CD4^+^ percentage (p = 0.007), CD8^+^ T-cell count (p < 0.05), and CD8^+^ percentage (p < 0.001), accompanied by a decrease in the CD4^+^/CD8^+^ ratio (p < 0.05). One-way ANOVA of Δ changes confirmed a significantly greater reduction in the CD4^+^/CD8^+^ ratio in ICT-MEL compared with both ICT-PLA and C-PLA (p < 0.05), along with greater increases in CD4^+^ percentage, CD8^+^ count, and CD8^+^ percentage. Following the intervention, CD4^+^ T-cell count was significantly higher in the ICT-PLA group compared with the C-PLA group (p < 0.05). B-cell percentage increased significantly from pre-to post-intervention only in the ICT-PLA group (p = 0.017), with *post hoc* analyses indicating a greater increase compared with C-PLA (p < 0.05). NK-cell counts and percentages remained unchanged across all groups. Notably, intermediate monocytes (CD14^++^CD16^+^) decreased significantly from pre-to post-intervention in the ICT-MEL group in both absolute count (p = 0.02) and percentage (p = 0.017), while classical and non-classical monocyte subsets showed no significant changes.

**Conclusion:**

ICT combined with melatonin supplementation was associated with favorable changes in immune cell populations in patients undergoing HD, including improved T-cell profiles and reduced intermediate monocyte subsets. These findings suggest potentially beneficial immunological adaptations and are consistent with a synergistic effect of exercise and MEL supplementation in this population.

## 1 Introduction

Patients undergoing hemodialysis (HD) experience significant immune dysfunction and hematological abnormalities, contributing to increased susceptibility to infections, chronic inflammation, and cardiovascular complications (Alhamawi et al., 2025). These patients often exhibit lymphopenia, characterized by decreased counts of CD4^+^ and CD8^+^ T lymphocytes, reduced natural killer (NK) cell activity, and dysregulated monocyte subsets, including increased proinflammatory monocytes and decreased classical monocytes (Alhamawi et al., 2025; Peraldi et al., 2013; Goyoneche et al., 2025). Additionally, anemia, leukocyte dysfunction, and impaired erythropoiesis are common hematological issues in this population (Portolés et al., 2021; Badura et al., 2024; Espi et al., 2020). These immune and hematologic impairments not only compromise patients’ quality of life but also increase morbidity and mortality. (Hanna et al., 2021).

Intradialytic concurrent training (ICT), which integrates both aerobic and resistance training during dialysis sessions, has emerged as a practical and effective strategy to improve functional capacity and overall health outcomes in HD patients (Marzougui et al., 2023a; Marzougui et al., 2024). Previous studies have reported that intradialytic exercise can modulate immune and inflammatory responses, characterized by reductions in circulating proinflammatory cytokines and improvements in lymphocyte function (Dungey et al., 2017). Additionally, such training may positively influence hematological parameters, including erythropoietic markers and lymphocyte counts, thereby contributing to better immune competence and anemia management (Dungey et al., 2017). Beyond its physiological effects, ICT offers unique logistical advantages by efficiently utilizing dialysis time, improving patient adherence, and minimizing barriers to regular physical activity (Marzougui et al., 2023a).

Melatonin (MEL), a pineal hormone with potent antioxidant, anti-inflammatory, and immunomodulatory properties, has received growing attention as an adjuvant therapy in patients with chronic kidney disease (CKD) and those undergoing HD (Marzougui et al., 2024; Marzougui et al., 2023b; Marzougui et al., 2021). Its actions extend beyond regulating circadian rhythms; MEL directly scavenges reactive oxygen and nitrogen species and upregulates endogenous antioxidant defenses such as superoxide dismutase and glutathione peroxidase (Tan et al., 2003; Hardeland, 2018). Moreover, it modulates immune cell function by enhancing lymphocyte proliferation, suppressing proinflammatory cytokines (e.g., TNF-α, IL-6), and regulating monocyte and macrophage activity (Carrillo-Vico et al., 2013). In HD patients, where oxidative stress and chronic inflammation are key contributors to immune dysregulation and anemia, MEL supplementation has been shown to attenuate lipid peroxidation, enhance antioxidant defenses, and reduce systemic inflammation (Markowska et al., 2023).

Recently, Marzougui et al. (2024) demonstrated that combining ICT with MEL supplementation synergistically reduced lipid peroxidation and systemic inflammation in HD patients, as evidenced by decreased malondialdehyde and C-reactive protein (CRP) levels, along with increased antioxidant capacity (Marzougui et al., 2024). In addition, Marzougui et al. conducted a crossover study assessing the acute immune effects of intradialytic exercise combined with MEL versus placebo (PLA) (Marzougui et al., 2021). Authors found that MEL prevented dialysis-induced decreases in NK cells and CD8^+^ T lymphocytes, increased CD4^+^ T-lymphocyte proportions, and reduced intermediate monocytes (CD14^++^CD16^+^), highlighting its potential to counteract HD-related immune disturbances (Marzougui et al., 2021). While these findings suggest that MEL can acutely modulate immune function during exercise, long-term adaptations to ICT combined with MEL supplementation remain poorly understood.

To our knowledge, no prior study has examined the combined long-term effects of ICT and MEL on hematological and immune cell subset adaptations in HD patients. Therefore, the present study investigates, for the first time, the impact of ICT combined with MEL supplementation on hematological and immune parameters, including CD4^+^ and CD8^+^ T lymphocytes, B cells, NK cells, and monocyte subsets.

## 2 Materials and methods

### 2.1 Sample size

The required sample size was calculated *a priori* using G*Power software (Faul et al., 2007), in accordance with the methodological recommendations of Beck et al. (Beck, 2013). The level of significance (α) was set at 0.05, and statistical power (1−β) was established at 0.95. Based on previous studies investigating the effects of MEL supplementation and intradialytic exercise on immune parameters, particularly changes in CD4^+^ and CD8^+^ T-cell responses (12,15), and on consensus within the research team, a large effect size (Cohen’s d = 0.8) was assumed. Under these parameters, a minimum of 21 participants was estimated to provide sufficient power to detect significant effects while minimizing the risk of a type II error.

### 2.2 Participants

**FIGURE 1:**
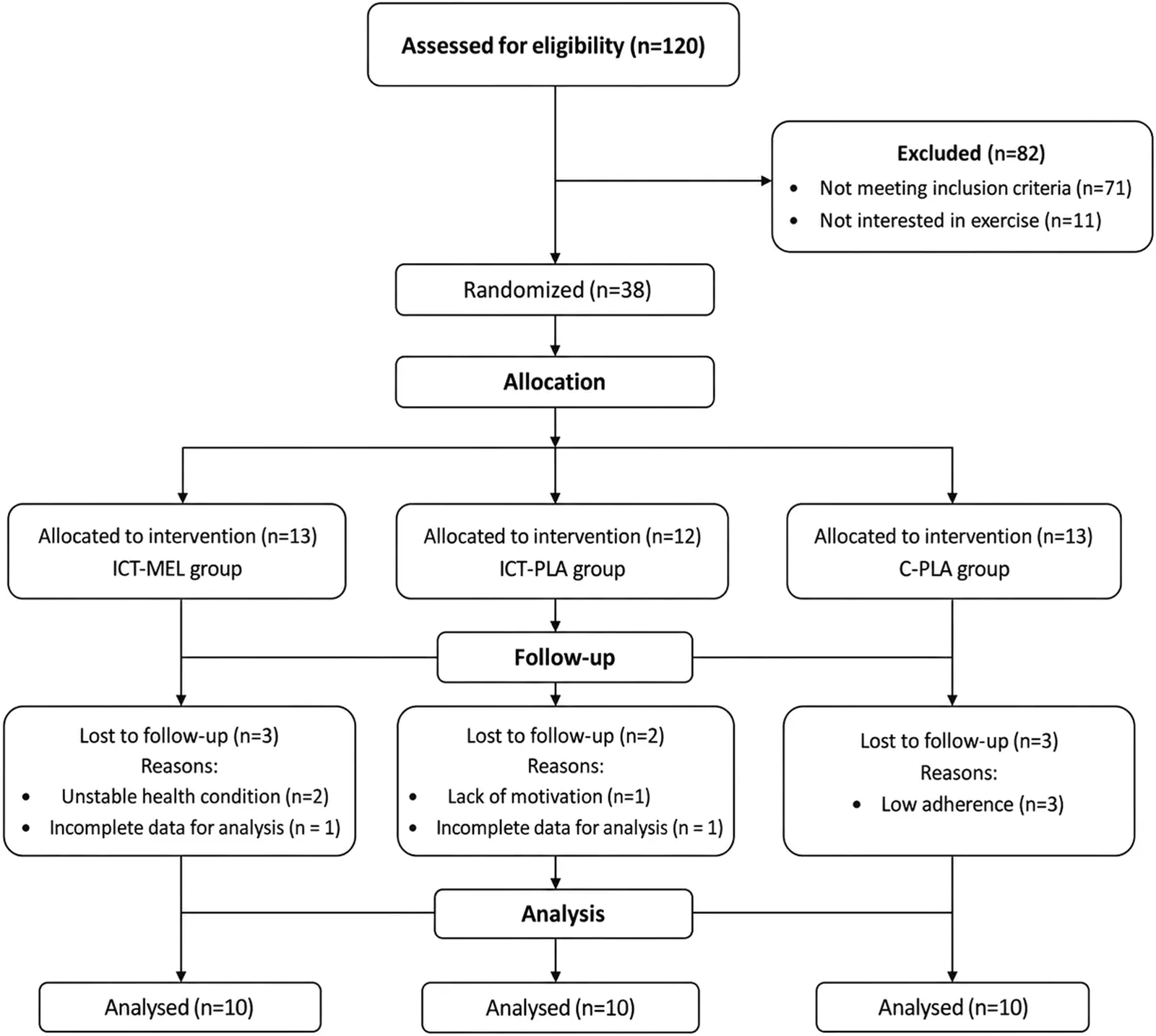
Flowchart of study participants.

Thirty-eight hemodialysis (HD) patients were recruited from the HD unit at Hedi Chaker University Hospital in Sfax, Tunisia. Participants were randomly allocated to one of three groups: ICT with melatonin (ICT-MEL, n = 13), ICT with placebo (ICT-PLA, n = 12), or control with placebo (C-PLA, n = 13). During the intervention period, eight participants did not complete the study. Consequently, 30 participants completed the study (n = 10 per group; mean age: 48.80 ± 10.62 years). All participants had been receiving maintenance HD treatment for at least 6 months, consisting of three 4-h sessions per week. Figure 1 illustrates the participant recruitment and allocation process, and Table 1 summarizes baseline demographic and clinical characteristics.

**TABLE 1:** Baseline characteristics by group allocation (n = 30).

Characteristic	ICT-MEL (n = 10)	ICT-PLA (n = 10)	C-PLA (n = 10)	P-value
Sex (male/Female)	7/3	6/4	3/7	​
Age (years)	50 ± 10.8	49.20 ± 13.17	47.20 ± 8.70	0.84
Height (cm)	166.60 ± 7.38	165 ± 9.36	162.70 ± 10.62	0.64
Dry weight (kg)	62.28 ± 6.46	62.49 ± 6.18	65.20 ± 14.36	0.76
BMI (kg/m^2^)	22.52 ± 2.89	23.22 ± 4.20	24.54 ± 3.99	0.48
Hemodialysis (months)	108.66 ± 16.84	113.33 ± 28.76	110 ± 24.37	0.90
Urea (mmol/L)	22.58 ± 5.23	21.46 ± 4.32	23,99 ± 3.69	0.45
Creatinine (µmol/L)	958.50 ± 159.57	936.20 ± 163.98	962.60 ± 157.04	0.73
Primary disease (n)				
• Glomerulonephritis	4	3	5	​
• Chronic interstitial nephritis	3	4	3	
• Family nephropathy	2	2	1	
• Uncertain	1	1	1	

Data are presented as mean ± SD. BMI: body mass index; ICT‐MEL: intradialytic concurrent exercise−melatonin; ICT‐PLA: intradialytic concurrent exercise−placebo; C‐PLA: control−placebo.

All patients had a functional arteriovenous fistula as vascular access. Exclusion criteria were: (i) participation in any structured exercise program within the previous 6 months; (ii) serious medical conditions requiring hospitalization or limiting physical activity (e.g., advanced cardiovascular disease); (iii) active infectious or inflammatory diseases; (iv) orthopedic limitations; (v) severe psychological or neurological disorders; (vi) use of antioxidant supplements (e.g., vitamin E, statins, or other agents with antioxidant properties); and (vii) use of psychoactive medications.

A dropout rate of approximately 15%–20% was anticipated based on the planned number of training sessions. All participants were fully informed about the study objectives and procedures and provided written informed consent prior to participation.

### 2.3 Study design

The study was conducted as a randomized, double-blind, placebo-controlled clinical trial. Participants assigned to ICT-MEL and ICT-PLA groups completed ICT sessions three times per week for a duration of 12 weeks. The ICT-MEL group received 3 mg of MEL (Jamieson Laboratories, Toronto, Canada), whereas participants in the ICT-PLA and C-PLA groups received a placebo (Galpharma Laboratories, Sfax, Tunisia) each evening, approximately 30 min before nocturnal sleep. The selected MEL dose was based on recommendations for HD patients by Koch et al. (Koch et al., 2009) and Russcher et al. (Russcher et al., 2013), with no significant adverse effects reported at this dosage. As previously described (Marzougui et al., 2023a), the timing of MEL administration followed the protocols established by Härtter et al. (Hartter et al., 2000) and Maldonado et al. (Maldonado et al., 2012).

Although participants in the C-PLA group were aware that they were not engaged in physical exercise, they remained blinded to whether they were receiving MEL or PLA. Similarly, participants in the ICT-MEL and ICT-PLA groups were unaware of their supplementation allocation, minimizing potential biases related to treatment expectations across all study groups. To reduce learning effects, participants in the ICT-MEL and ICT-PLA groups were familiarized with the lower-limb cycle ergometer, resistance exercises (including knee extension, hip abduction, and flexion), and the modified Rating of Perceived Exertion (RPE) Borg scale (Borg, 1982). Anthropometric measurements, including height and body mass, were obtained using a semi-analytical balance, and body mass index (BMI) was calculated as weight (kg) divided by height squared (m^2^).

The study protocol was approved by the South Institutional Committee of Personal Protection (CPP) in Sfax, Tunisia (No. 0059/2017) and was conducted in accordance with the ethical principles outlined in the Declaration of Helsinki (2024) (Association, 2025). The trial was also registered in the Pan African Clinical Trials Registry under the registration number PACTR202005770507528.

### 2.4 Concurrent training program

Participants in the ICT-MEL and ICT-PLA groups performed an ICT program during the first 2 hours of their HD sessions under physician supervision. Each session combined endurance and resistance exercises. The endurance component began with a 5-min warm-up, followed by 15 min of cycling on a lower-limb cycle ergometer (Everfit Welly-M, Pozzolo Formigaro, Alessandria, Italy), positioned in front of each participant. Cycling duration was progressively increased by 5 min after the first week and by 10 min after the second week, reaching 30 min by the end of the first 2 weeks. Subsequently, session duration was extended by 5%–10% each week, culminating in 60 min by the end of the 12-week intervention. Exercise intensity was prescribed and monitored using heart rate (HR), targeting 50%–60% of each participant’s maximum HR (HRmax = 207–0.7 × age) (Gellish et al., 2007). Given that many participants were taking beta-blockers, the modified Borg RPE scale (Borg, 1982) was also used to assess exercise intensity subjectively. The scale ranges from 0 (no effort) to 10 (maximal effort), and participants were instructed to cycle at a pace corresponding to an RPE of 3–4, indicating moderate to somewhat hard exertion.

Resistance exercises included knee extension, hip abduction, and hip flexion, with initial weights determined through a three-repetition maximum (3-RM) test (Marzougui et al., 2023a). Participants performed 2–3 sets of 10 repetitions at approximately 60% of their 3-RM using ankle weights, with loads progressively increased once participants were able to complete three sets with proper form. Rest periods of 1 min were allowed between sets and 3 min between exercises. During training sessions, exercise intensity and vital signs, including blood pressure, HR, and oxygen saturation, were monitored every 5 min. Each session concluded with lower-limb stretching exercises targeting the hamstrings, hip adductors, hip abductors, tibialis anterior, gastrocnemius, and soleus. The exercise regimen was paused if participants experienced any of the following symptoms: chest pain, arrhythmias, shortness of breath, nausea, muscle pain or cramps, hypotension or hypertension episodes, or a high RPE score on the modified Borg scale. Participants in the C-PLA group continued their standard HD sessions without engaging in ICT.

### 2.5 Blood sampling and analysis

Blood samples were collected at baseline and after 12 weeks, with all collections performed at the same time of day (between 07:00 and 07:30 AM) during midweek. Participants were instructed to refrain from exercise for 24 h prior to sampling. Ten milliliters (mL) of venous blood were drawn from the antecubital vein into three tubes: 4 mL containing heparin, 3.5 mL containing K2EDTA, and 2.5 mL without anticoagulant. Samples in tubes without anticoagulant and those containing heparin were centrifuged for 10 min at 2,500 rpm and then aliquoted. Serum and plasma were stored at −80 °C for subsequent analyses. Tubes containing K2EDTA were used for hematology and immunophenotyping, with analysis performed within 3 hours of collection.

Hematological parameters, including complete blood counts and differential white blood cell counts, were measured using an automated cell counter. Flow cytometric immunophenotyping was employed to assess lymphocyte and monocyte subsets using directly labeled monoclonal antibodies. T lymphocyte subsets (CD4^+^, CD8^+^), natural killer (NK) cells (CD16^+^/CD56^+^), B lymphocytes (CD19^+^), and monocyte subsets (classical CD14^++^CD16^−^, intermediate CD14^++^CD16^+^, and non-classical CD14^+^CD16^++^) were quantified following the manufacturers’ instructions. For each participant, two 100 μL whole-blood specimens were labeled with specific antibody panels: the first tube contained BD PerCP-Cy5.5/CD45, CD3/FITC, PE-Cy7/CD4, APC-Cy7/CD8, PE/CD16–CD56, and APC/CD19 for lymphocyte phenotyping, while the second tube contained PE/CD16 and FITC/CD14 for monocyte subsets analysis. Samples were incubated for 15 min at room temperature in the dark with the fluorochrome-conjugated antibodies. Erythrocytes were subsequently lysed using 2 mL of BD FACS Lysing Solution according to the manufacturer’s instructions, and remaining cells were washed and suspended in cellwash buffer.

Flow cytometric acquisition was performed on a FACS Canto II, collecting 10,000–50,000 events per sample. Lymphocyte subsets were identified using forward and side scatter gating, with the lymphocyte population verified for purity using the CD45 marker. Data were analyzed using Diva software, and lymphocyte subpopulations were classified based on their specific immunophenotypic markers.

### 2.6 Statistical analysis

Data are presented as mean ± standard deviation (SD) in tables and figures. The Shapiro–Wilk test and Levene’s test were used to verify the assumptions of normality and homogeneity of variance, respectively. Differences in baseline group characteristics were examined using one-way ANOVA for normally distributed variables or the Kruskal–Wallis test for non-normally distributed variables.

A two-way repeated-measures ANOVA [Groups (ICT-MEL vs. ICT-PLA vs. C-PLA) × Time (pre-vs. post-intervention)] was applied to evaluate within-subject changes over time and time × group interactions. For normally distributed variables, paired-samples t-tests were used to assess within-group pre-to post-intervention changes. Practical significance was assessed by calculating effect sizes, using partial eta-squared (ηp^2^) for ANOVA results. A one-way ANOVA on delta changes (Δ = post-intervention value − pre-intervention value) was conducted for normally distributed between-group comparisons. When significant effects were identified in the ANOVA analyses, pairwise post hoc comparisons were performed using the Bonferroni adjustment.

For variables that were not normally distributed, Wilcoxon signed-rank tests were used for within-group comparisons, and Kruskal–Wallis tests were applied to Δ changes for between-group comparisons. Post hoc analyses for non-parametric tests were performed using the Mann–Whitney U test with Bonferroni correction. To account for multiple testing across the immunological outcomes, the Benjamini–Hochberg false discovery rate (FDR) procedure was applied with a false discovery rate set at 5% (Benjamini et al., 1995). Adjusted p-values were calculated for the immunophenotyping outcomes, and statistical significance was determined based on the FDR-adjusted values.

The threshold for statistical significance was set at p < 0.05. All analyses were performed using IBM SPSS Statistics version 27.0 (IBM Corp., Armonk, NY, United States).

## 3 Results

### 3.1 Hematology

No significant differences were observed in white blood cell, neutrophils, red blood cell, hemoglobin, hematocrit, neutrophil-to-lymphocyte ratio, lymphocyte or monocytes following the intervention across all groups (Table 2).

**TABLE 2:** Effects of intradialytic concurrent training and melatonin supplementation on hematological parameters.

Variables	ICT-MEL (n = 10)			ICT-PLA (n = 10)			C-PLA (n = 10)		
	Pre	Post	P-value	Pre	Post	P-value	Pre	Post	P-value
WBC (×10^3^/µL)	6.02 ± 1.82	5.63 ± 1.45	0.38	7.30 ± 3.36	6.60 ± 2.22	0.46	6.81 ± 2.13	6.22 ± 1.89	0.40
RBC (×10^6^/µL)	3.28 ± 0.94	3.35 ± 0.77	0.68	3.45 ± 0.62	3.40 ± 0.42	0.72	3.28 ± 0.49	3.13 ± 0.59	0.79
LY (×10^3^/µL)	1.24 ± 0.38	1.22 ± 0.35	0.85	1.61 ± 0.44	1.58 ± 0.59	0.83	1.41 ± 0.48	1.29 ± 0.38	0.20
MO (×10^3^/µL)	0.49 ± 0.24	0.50 ± 0.15	0.66	0.93 ± 1.02	0.49 ± 0.20	0.38	0.51 ± 0.19	0.53 ± 0.28	0.67
NE (×10^3^/µL)	4.13 ± 1.70	3.66 ± 1.12	0.57	4.35 ± 2.71	4.21 ± 1.59	0.15	4.60 ± 1.57	4.06 ± 1.53	0.28
HGB (g/dL)	10.16 ± 2.37	10.33 ± 2.06	0.76	10.70 ± 1.78	10.33 ± 1.26	0.59	10.35 ± 1.25	9.97 ± 1.72	0.91
HCT (%)	30.54 ± 7.99	30.99 ± 6.36	0.83	32.17 ± 5.39	30.94 ± 3.83	0.57	30.79 ± 3.87	29.25 ± 5.24	0.76
N/L ratio	3.44 ± 1.29	3.17 ± 1.17	0.28	2.91 ± 2.01	2.80 ± 0.92	0.44	3.32 ± 0.63	3.28 ± 1.10	0.76

Data are presented as mean ± SD. P-values were derived from paired-samples t-tests or Wilcoxon signed-rank tests, as appropriate. ICT-MEL: intradialytic concurrent exercise-melatonin; ICT-PLA: intradialytic concurrent exercise-placebo; C-PLA: control-placebo; Pre and Post correspond to data collection before and after 12 weeks of intervention. WBC: white blood cell, RBC: red blood cell, LY: lymphocytes, MO: monocytes, NE: neutrophils, HGB: hemoglobin, HCT: hematocrit, N/L Ratio: neutrophil-to-lymphocyte ratio.

### 3.2 Lymphocyte subsets (CD4^+^, CD8^+^ T Cells, B Cells, and NK Cells)

A significant group effect was observed for CD4^+^ T-cell count (F_(2,27)_ = 3.92, p < 0.05, ηp^2^ = 0.22). Post hoc analysis indicated that CD4^+^ T-cell count was significantly higher in the ICT-PLA group compared with the C-PLA group following the intervention (p = 0.031, FDR-adjusted q = 0.027).

For CD4^+^%, the Wilcoxon signed-rank test indicated a significant increase after the intervention in the ICT-MEL group (W = 54.00, Z = 2.70, p = 0.007). Analysis of Δ changes using the Kruskal–Wallis test showed a significant group effect (p < 0.05), with *post hoc* comparisons revealing that ICT-MEL exhibited a greater increase than both ICT-PLA (p = 0.016, FDR-adjusted q = 0.032) and C-PLA (p = 0.030, FDR-adjusted q = 0.030) from pre-to post-intervention.

CD8^+^ T-cell count increased significantly following the intervention in the ICT-MEL group (W = 47.00, Z = 1.98, p < 0.05). The Kruskal–Wallis test performed on Δ changes also showed a significant group effect (p < 0.05), and *post hoc* analyses confirmed higher Δ values in ICT-MEL compared with ICT-PLA (p = 0.018, FDR-adjusted q = 0.024) and C-PLA (p = 0.012, FDR-adjusted q = 0.031) from pre-to post-intervention.

A repeated-measures ANOVA for CD8^+^% revealed significant effects of time (F_(1,27)_ = 24.72, p < 0.001, ηp^2^ = 0.47) and time × group interaction (F_(2,27)_ = 27.93, p < 0.001, ηp^2^ = 0.67). Paired-samples t-tests indicated a significant post-intervention increase in CD8^+^% within the ICT-MEL group (p < 0.001). One-way ANOVA of Δ changes further confirmed a greater increase in CD8^+^% for ICT-MEL compared with ICT-PLA (p < 0.001, FDR-adjusted q = 0.008) and C-PLA (p < 0.001, FDR-adjusted q = 0.004) from pre-to post-intervention.

For the CD4^+^/CD8^+^ ratio, repeated-measures ANOVA showed a significant time × group interaction (F_(2,27)_ = 4.99, p < 0.05, ηp^2^ = 0.27). Paired-samples t-tests revealed a significant post-intervention decrease in the ICT-MEL group (p < 0.05), and one-way ANOVA of Δ changes indicated a greater reduction in ICT-MEL compared with ICT-PLA (p = 0.023, FDR-adjusted q = 0.026) and C-PLA (p = 0.047, FDR-adjusted q = 0.038) from pre-to post-intervention.

No significant differences were observed for B-cell count. However, for B-cell percentage, the Wilcoxon signed-rank test showed a significant increase after the intervention in the ICT-PLA group (W = 51.00, Z = 2.39, p = 0.017). Analysis of Δ changes using the Kruskal–Wallis test indicated a significant group effect (p < 0.05). Post hoc tests revealed that ICT-PLA increased significantly compared with C-PLA (p = 0.016, FDR-adjusted q = 0.026), while the C-PLA group showed a non-significant trend toward a decrease (W = 11.00, Z = −1.68, p = 0.09) from pre-to post-intervention. No significant differences were observed in NK-cell count, NK%, NKT-cell count, or NKT% (Table 3).

**TABLE 3:** Effects of intradialytic concurrent training and melatonin supplementation on lymphocyte subsets.

Variables	ICT-MEL (n = 10)			ICT-PLA (n = 10)			C-PLA (n = 10)		
	Pre	Post	Δ Change (median [IQR])	Pre	Post	Δ Change (median [IQR])	Pre	Post	Δ Change (median [IQR])
CD4^+^ (cells/µL)	524.88 ± 140.05	619.38 ± 146.78	152.15 [-31.8–250]	717.32 ± 275.32	723.8 ± 302.14^≠^	−15.75 [-58.8–71.2]	507.02 ± 164.38	487.16 ± 113.96	−10.95 [−74.6–107.6]
CD4^+^ (%)	43.43 ± 7.86	51.6 ± 5.05**	9.25 [5.9–10.9]^¶, ¥^	43.87 ± 9.05	45.43 ± 4.62	0.64 [-3.9–8.9]	36.73 ± 6.78	39.16 ± 9.61	2.20 [0.50–6.50]
CD8^+^ (cells/µL)	357.07 ± 167.71	490.91 ± 159.98*	143.3 [74.1–224.3]^¶, ¥^	449.42 ± 159.66	425.82 ± 179.3	−76.2 [-109.2–55.6]	437.30 ± 216.65	401.54 ± 184.04	−46.2 [-101–29.2]
CD8^+^ (%)	28.03 ± 5.57	39.9 ± 2.90***	12.9 [8.2−16.7]^¶¶, ¥¥^	28.16 ± 6.9	27.33 ± 7.28	−1.25 [-2.8–1.4]	29.96 ± 5.42	30.34 ± 7.61	1.2 [-2.4–1.4]
CD4^+^/CD8^+^ ratio	1.62 ± 0.51	1.30 ± 0.17*	−0.24 [-0.68–0.02]^¶, ¥^	1.64 ± 0.56	1.79 ± 0.58	0.03 [-0.16–0.3]	1.27 ± 0.38	1.37 ± 0.50	0 [-0.04–0.34]
B-cell count (cells/µL)	88.73 ± 56.58	103.42 ± 68.70	16.35 [-27.5–42.8]	122.92 ± 77.22	146.25 ± 87.23	4.85 [-10.9–66.9]	106.04 ± 51.16	87.01 ± 51.75	17.95 [-38–3.9]
B cells (%)	6.76 ± 3.61	7.86 ± 3.68	1.55 [0.2–3.3]	7.31 ± 3.59	9.28 ± 5.09*	1.35 [0.4–3.4]^¥^	7.76 ± 3.79	6.59 ± 3.32	−1.8 [-3.1–1]
NK-cell count (cells/µL)	244.62 ± 128.32	222.87 ± 102.18	−9.45 [-56.9–6.8]	288.82 ± 159.18	260.67 ± 122.13	−12 [-60.6–73.5]	318.11 ± 135.13	294.29 ± 140.42	−23.8 [-81.2–41.7]
NK cells (%)	19.76 ± 8.33	18.36 ± 6.46	−2.1 [-3.2–0.10]	18.13 ± 9.75	16.34 ± 4.97	−0.35 [-3.8–1.8]	22.72 ± 7.20	22.06 ± 8.97	−1.7 [-7–3.6]
NKT-cell count (cells/µL)	133.53 ± 111.63	101.37 ± 81.20	−2.2 [-82.8–9.5]	145.77 ± 107.03	137.59 ± 119.44	−18.7 [-42.8–12.2]	114.33 ± 89.32	94.59 ± 71.02	−7.4 [-59.5–2.2]
NKT cells (%)	9.61 ± 5.70	8 ± 5.03	−0.25 [-1.6–0.2]	9.18 ± 6.13	8.42 ± 5.46	−1.45 [-2.9–0.1]	7.79 ± 4.98	7.16 ± 4.74	−0.15 [-1.7–0.5]

Pre- and post-intervention values are presented as mean ± SD, whereas Δ change values are presented as median [interquartile range (IQR)]. P-values were derived from paired-samples t-tests or Wilcoxon signed-rank tests, as appropriate. ICT-MEL: intradialytic concurrent exercise-melatonin; ICT-PLA: intradialytic concurrent exercise-placebo; C-PLA: control-placebo. Pre and post correspond to measurements obtained before and after 12 weeks of intervention. CD4^+^ and CD8^+^ T lymphocytes represent helper and cytotoxic T-cell subsets, respectively. B cells were identified as CD19^+^ lymphocytes; natural killer (NK) cells as CD16^+^/CD56^+^ lymphocytes; and natural killer T (NKT) cells as CD3^+^CD16^+^/CD56^+^ lymphocytes. Absolute cell counts are expressed as cells/µL and relative values as percentages (%) of total lymphocytes. The CD4^+^/CD8^+^ ratio was calculated from absolute counts.

*p < 0.05, **p < 0.01, ***p < 0.001 vs. pre-intervention within the same group.

^¶^p < 0.05, ^¶¶^p < 0.001 vs. ICT-PLA (Δ change).

^¥^ p < 0.05, ^¥¥^ p < 0.001 vs. C-PLA (Δ change).

^≠^ p < 0.05 for the comparison between ICT-PLA and C-PLA at post-intervention.

### 3.3 Monocyte subsets

**FIGURE 2:**
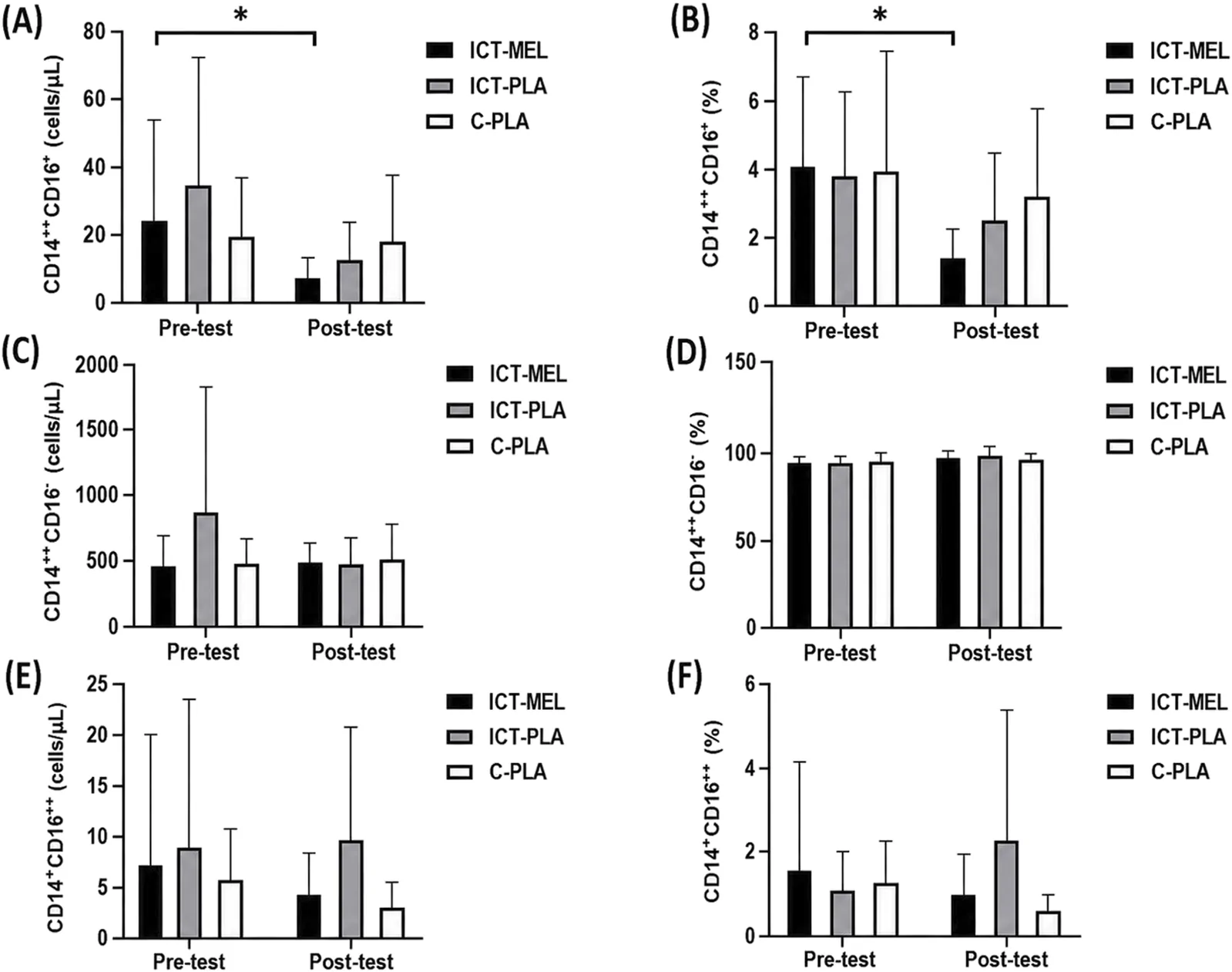
Effect of intradialytic concurrent training and melatonin supplementation on monocyte subsets in hemodialysis patients. Data are presented as mean ± SD. P-values were derived from paired-samples t-tests or Wilcoxon signed-rank tests, as appropriate. ICT-MEL: intradialytic concurrent exercise-melatonin; ICT-PLA: intradialytic concurrent exercise-placebo; C-PLA: control-placebo. Pre and Post correspond to measurements obtained before and after 12 weeks of intervention. CD14^++^CD16^+^ **(A,B)**, CD14^++^CD16^−^ **(C,D)**, and CD14^+^CD16^++^ **(E,F)** correspond to intermediate, classical, and non-classical monocyte subsets, respectively. Absolute monocyte counts are expressed as cells/µL, and subset distributions are expressed as percentages (%) of total monocytes. *p < 0.05 indicates a significant difference between Pre and Post.

For CD14^++^CD16^+^, the Wilcoxon signed-rank test indicated a significant decrease after the intervention in the ICT-MEL group (W = 5.00, Z = −2.29, p = 0.02), while the ICT-PLA group showed a non-significant trend toward a decrease (W = 10.00, Z = −1.78, p = 0.074) (Figure 2A). Similarly, the percentage of CD14^++^CD16^+^ decreased significantly in the ICT-MEL group after the intervention (W = 4.00, Z = −2.39, p = 0.017) (Figure 2B).

No significant effects were observed for classical monocytes (CD14^++^CD16^−^) or non-classical monocytes (CD14^+^CD16^++^), either in absolute number or percentage, across groups from pre-to post-intervention (Figures 2C–F).

## 4 Discussion

The present randomized, double-blind, placebo-controlled trial is the first to investigate the long-term combined effects of ICT and MEL supplementation on hematological parameters and immune cell subset adaptations in HD patients. The main findings demonstrate that ICT combined with MEL induces favorable immunological adaptations, particularly in T-lymphocyte subsets and monocyte phenotypes, without significantly altering global hematological parameters. These findings extend previous evidence on the acute immunomodulatory effects of intradialytic exercise and MEL by demonstrating sustained immune adaptations following a 12-week intervention (Marzougui et al., 2024; Marzougui et al., 2021).

Consistent with previous reports, no significant changes were observed in hematological parameters, including hemoglobin, hematocrit, and total leukocyte count, across groups from pre-to post-intervention (Lin et al., 2021). This finding aligns with evidence that erythropoiesis in HD patients is tightly regulated and predominantly influenced by inflammation, iron dysregulation, and resistance to erythropoietin, which may limit hematological adaptations to short-to-moderate exercise interventions (Portolés et al., 2021; Badura et al., 2024; Hanna et al., 2021). In line with this interpretation, intradialytic exercise programs have been shown to exert anti-inflammatory immune effects without significantly altering hemoglobin or hematocrit levels in HD patients (Dungey et al., 2017). Importantly, the maintenance of hematological stability is clinically relevant in this population, given the high prevalence of anemia and the vulnerability of erythropoiesis to dialysis-related stressors (Portolés et al., 2021; Badura et al., 2024; Espi et al., 2020).

A key finding of this study is the improvement in T-lymphocyte profiles following ICT combined with MEL supplementation. The ICT-MEL group exhibited significant increases in CD4^+^ and CD8^+^ T-cell counts and percentages, accompanied by a reduction in the CD4^+^/CD8^+^ ratio from pre-to post-intervention. These adaptations suggest favorable alterations in T-lymphocyte profiles, which are commonly impaired in HD patients due to uremia-induced immune exhaustion, oxidative stress, and chronic inflammation. (Alhamawi et al., 2025; Peraldi et al., 2013; Goyoneche et al., 2025; Espi et al., 2020). Regular intradialytic exercise has previously been shown to promote a more anti-inflammatory leukocyte profile and improve lymphocyte responsiveness in HD patients (Dungey et al., 2017). The present findings indicate that MEL supplementation may potentiate these exercise-induced immune benefits. Although MEL supplementation may have contributed to the observed immunological adaptations, its specific contribution relative to intradialytic concurrent training warrants further investigation.

MEL exerts multiple immunoregulatory actions, including stimulation of T-cell proliferation, modulation of cytokine production, and protection of immune cells against oxidative damage (Tan et al., 2003; Hardeland, 2018; Carrillo-Vico et al., 2013). In HD patients, oxidative stress and persistent inflammation are major contributors to immune dysfunction and lymphopenia (Peraldi et al., 2013; Espi et al., 2020; Markowska et al., 2023). The observed improvements in CD4^+^ and CD8^+^ T-cell parameters in the ICT-MEL group are therefore likely attributable to the synergistic interaction between exercise-induced immune activation and MEL-mediated antioxidant and anti-inflammatory effects (Marzougui et al., 2024; Kruk et al., 2021). These findings are consistent with previous acute studies demonstrating that MEL ingestion before intradialytic exercise may attenuate dialysis-induced reductions in CD8^+^ T lymphocytes and enhance CD4^+^ T-cell proportions (Marzougui et al., 2021), in agreement with other studies reporting immunomodulatory effects of MEL on T-cell responses (Carrillo-Vico et al., 2013; Calvo et al., 2013).

Consistent with the present findings, previous analyses from the same randomized controlled trial demonstrated favorable effects of the intervention on oxidative stress and inflammatory markers (Marzougui et al., 2024). However, these variables were not evaluated in relation to the immune cell outcomes reported in the present study. Therefore, although these mechanisms may have contributed to the observed immunological adaptations, their specific role in mediating the immune cell responses warrants further investigation.

Sleep quality represents another potential pathway through which melatonin may influence immune regulation. However, sleep-related outcomes were not included among the study endpoints and should be considered in future investigations.

However, these findings should be interpreted with caution, as the present study assessed phenotypic immune adaptations rather than functional immune outcomes. Therefore, although the observed changes in T-cell subsets suggest favorable immunological adaptations, further studies incorporating functional immune assessments and clinical endpoints are needed to determine their functional and clinical significance.

It is important to note that although the CD4^+^/CD8^+^ ratio decreased (from 1.62 ± 0.51 to 1.30 ± 0.17) in the ICT-MEL group, it remained within the normal physiological range (Louati et al., 2019), suggesting a shift in immune balance without pathological significance. This reduction likely reflects a physiological adaptation to chronic exercise. The greater proportional expansion of CD8^+^ T cells may reflect an adaptive response to chronic exercise, a phenomenon that has been reported following repeated exercise stimuli (Campbell and Turner, 2018).

In contrast, no significant long-term changes were observed in NK-cell number or percentage. This finding contrasts with earlier acute studies reporting protective effects of MEL on NK-cell counts during single intradialytic exercise sessions (Marzougui et al., 2021), as well as with previous reports highlighting the short-term immunomodulatory effects of MEL on NK-cell activity (Hardeland, 2018; Carrillo-Vico et al., 2013). The absence of sustained NK-cell adaptations may reflect the complex regulation of innate immunity in HD patients, where chronic uremic toxicity, altered cytokine signaling, and dialysis-related oxidative stress can limit NK-cell plasticity (Peraldi et al., 2013; Espi et al., 2020).

Regarding B cells no significant changes were observed in absolute cell counts, consistent with previous reports describing persistent B-cell dysregulation in HD patients (Alhamawi et al., 2025; Goyoneche et al., 2025). To date, no studies have specifically examined B cells responses to exercise interventions in HD patients; evidence from other populations indicates that exercise induces variable and often modest effects on B-cell number and function, with inconsistent findings across studies (Campbell and Turner, 2018). However, the increase in B-cell percentage observed in the ICT-PLA group may reflect relative shifts in lymphocyte distribution rather than true expansion, underscoring the need for cautious interpretation of proportional data in populations with altered leukocyte dynamics.

Importantly, ICT combined with MEL supplementation resulted in a significant reduction in CD14^++^CD16^+^ monocytes. This subset is strongly associated with proinflammatory activity, endothelial dysfunction, and cardiovascular risk in HD patients (Alhamawi et al., 2025; Goyoneche et al., 2025; Espi et al., 2020). Elevated levels of CD14^++^CD16^+^ are a hallmark of chronic inflammation and have been linked to increased morbidity and mortality in end-stage kidney disease (Espi et al., 2020). In HD patients, exercise training has been shown to shift monocyte phenotypes toward less inflammatory profiles (Dungey et al., 2017), while MEL suppresses monocyte activation and inflammatory cytokine production (Carrillo-Vico et al., 2013; Markowska et al., 2023). The selective reduction of CD14^++^CD16^+^ observed in the ICT-MEL group may therefore represent a potentially beneficial anti-inflammatory adaptation. Although this finding is consistent with a less pro-inflammatory immune phenotype, additional studies incorporating functional inflammatory assessments are needed to clarify its clinical relevance.

Limitations of the present study should be acknowledged. Although the sample size calculation indicated adequate statistical power for the primary outcomes (GPower, α = 0.05, power = 0.95, effect size = 0.8), the final sample size after attrition (n = 10 per group) may limit the generalizability of the findings and the detection of smaller effects. Moreover, the results are based on immune cell counts and phenotypic markers. Their functional and clinical relevance should therefore be interpreted with caution and confirmed in future studies using functional immune assays and clinically relevant outcomes. Sleep quality and sleep-related outcomes were not assessed and should be considered in future investigations, given the known physiological effects of MEL. Furthermore, exercise intensity was prescribed using an age-predicted HRmax equation that has not been specifically validated in HD patients, although exercise intensity was also monitored using the modified Borg RPE scale. In addition, concomitant medications were not systematically evaluated for potential interactions affecting MEL pharmacokinetics, which may have contributed to interindividual variability in MEL bioavailability. Larger studies with predefined primary immune endpoints and comprehensive functional immune assessments are warranted to confirm and extend the present findings.

In conclusion, this study demonstrates that a 12‐week program of ICT combined with MEL supplementation was associated with favorable changes in several immune cell populations in patients undergoing HD. These findings suggest potentially beneficial immunological adaptations and are consistent with a synergistic effect of exercise and MEL supplementation on immune cell adaptations in this population. However, further studies incorporating functional immune assessments, clinical endpoints, and sleep-related outcomes are needed to clarify the underlying mechanisms and clinical relevance of these adaptations.

## Data Availability

The raw data supporting the conclusions of this article will be made available by the authors, without undue reservation.
